# Efficacy and safety of tacrolimus in older adults with ulcerative colitis: a retrospective study

**DOI:** 10.1186/s12876-023-03089-4

**Published:** 2024-01-08

**Authors:** Ayumi Ito, Syun Murasugi, Teppei Omori, Shinichi Nakamura, Katsutoshi Tokushige

**Affiliations:** Department of Gastroenterology, Tokyo Women’s Medical University, 8-1 Kawada-cho, Shinjuku-ku, Tokyo, 162-8666 Japan

**Keywords:** Ulcerative colitis, Aged, Tacrolimus, Kidney diseases

## Abstract

**Background/aims:**

The prevalence of ulcerative colitis (UC) has been increasing, also in older adults. Here, we retrospectively compared the efficacy and safety of tacrolimus (TAC) in older and younger patients with UC.

**Methods:**

We included younger (age < 65 years; *n* = 116) and older patients (age ≥ 65 years; *n* = 21) with UC who received TAC from April 2009 through December 2022(mean follow-up, 1230 ± 175 days) and achieved remission. Evaluations included age at onset, laboratory values, estimated glomerular filtration rate (eGFR), use of 5-aminosalicylic acid (5-ASA), biological experience, colonoscopy scores, remission at 1 month after treatment initiation, and adverse events. Treatment duration and renal function were assessed in patients with follow-up data (younger patients, *n* = 110; older patients, *n* = 19).

**Results:**

Older patients had a higher age at onset and treatment initiation but less 5-ASA use and biological experience. Before treatment, hemoglobin, albumin, and eGFR were significantly lower in the older group and CRP was significantly higher. The remission rate was 80.1% in the younger group and 66.6% in the older group (*P* = 0.1862). Adverse events were similar in both groups. The older group had a shorter treatment duration and significantly less change in renal function at all time points.

**Discussion:**

Rates of TAC-induced remission and adverse events were similar in older and younger adults with UC.

**Conclusion:**

TAC can be used safely in elderly patients with moderate to severe UC with careful monitoring.

## Introduction

Ulcerative colitis (UC), a chronic inflammatory bowel disease, is common among young people but, because it is a life-long disease, is also found in older adults. In addition, some patients develop UC when they are older. In fact, as the number of UC patients has increased, so has the number of older UC patients [1]. Understanding the characteristics and treatment of UC in older adults is particularly important in Japan because it is one of the most rapidly aging societies in the world. In older UC patients, more attention must be paid to general condition, medical history, and drug adverse events than in younger patients [2]. Moreover, the mortality rate appears to be higher in older patients with more severe UC [1].

Among various ulcerative colitis treatments, tacrolimus (TAC), a calcineurin inhibitor, has been shown to be effective in the treatment of severe and refractory ulcerative colitis [3].

In our hospital, infliximab or tacrolimus is the treatment of choice for steroid-resistant ulcerative colitis. If underlying diseases and infections are checked and cardiac complications such as heart failure or tuberculosis cannot be ruled out, tacrolimus should be used unless renal dysfunction is present. To date, limited information is available on older patients with UC. For example, only case reports have been published on the treatment of severe UC with the TAC [4]. Therefore, we retrospectively compared patient characteristics and TAC efficacy, safety, and impact on renal function in younger and older patients with UC who achieved remission with TAC.

## Methods

### Study design and participants

This was a retrospective cohort study. Participants were patients who received TAC from April 2009 through June 2022 (*n* = 137). On the basis of age at treatment initiation, patients were stratified into a younger (age < 65 years; *n* = 116) and older group (age ≥ 65 years; *n* = 21). The following clinical background information was collected: sex, age at disease onset, disease duration, disease area, concomitant drugs, Biological experience, Lichtiger clinical activity index (CAI) at admission, hemoglobin (Hb), albumin (Alb), creatinine (Cr), C-reactive protein (CRP), estimated glomerular filtration rate (eGFR).

The Mayo endoscopic subscore and UCEIS were used for the endoscopic score [5, 6].

The time to reach the target TAC trough concentration, length of hospital stay, underlying disease requiring long-term treatment and follow-up, Charlson Comorbidity Index (CCI) [7], remission rate (%) and TAC-related adverse events after 1 month of TAC administration were also examined. The target trough for TAC was set at a high concentration (10-15 ng/ml) for the first 2-3 weeks of TAC. Subsequently, the concentration was lowered to a lower level (5-10 ng/ml). Tacrolimus was administered orally twice daily. After administration, trough concentrations were adjusted by blood sampling [3].

In patients who were followed up after TAC treatment initiation (younger patients, *n* = 110; older patients, *n* = 19), the following parameters were evaluated: TAC treatment duration; renal function at 2, 4, 12, and 24 weeks after initiation of TAC treatment and 48 weeks after discontinuation of treatment; and changes in renal function (pre-treatment eGFR and post-treatment eGFR).

Remission was defined as a CAI less than or equal to 4 [4]. Concomitant diseases were assessed with the CCI without age [8].

### Statistical analysis

Results are presented as either number of patients or mean ± standard deviation (SD). The Mann-Whitney and chi-square tests were used for between-group comparisons. Maintenance rates were determined by the Kaplan-Meier estimator and compared by the log-rank test. A *P* value of less than 0.05 was considered significant. Statistical analysis was performed with JMP Pro16 [Statistical Discovery, SAS].

## Results

### Clinical characteristics

**Fig. 1:**
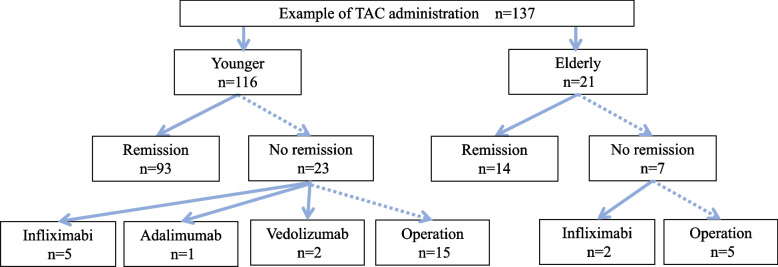
Flow chart after TAC administration

A flowchart of the treatment process of this study is shown below (Fig. 1).

The older patient group was significantly older at the start of treatment and at the onset of disease, and had significantly less frequent use of 5-ASA and significantly less experience with biologic agents. Pre-treatment Hb, Alb, Cr, and eGFR were also significantly lower in older patients. In contrast, pre-treatment CRP was significantly higher in the older patient group. According to CAI scores, all patients had moderate or severe UC. No significant difference was observed in CAI, colonoscopy scores (Mayo Score and UCEIS), time to reach TAC trough concentration, or hospitalization period. On the other hand, there was a significant difference in the presence of underlying diseases: apart from liver diseases, all other diseases, including cancer, were significantly more common in the older patient group (*P* < 0.05) (Table 1).

**Table 1:** Clinical characteristics of the total UC patient series

	Younger (< 65 yrs. old) *n* = 116	Elderly (≥65 yrs. old) *n* = 21	*p*-value
Gender, male:female	76:40	9:12	0.052
Age (yrs)	39.4 ± 11.7	68.9 ± 6.2	< 0.0001
Age at onset (yrs)	32 ± 12.6	59.8 ± 11.6	< 0.0001
Duration of disease	7.9 ± 7.6	9.4 ± 10.1	0.923
Site involved left-sided colitis/total colitis	31 / 85	3 /18	0.202
Concomitant drugs:			
5-ASA	102 (87.9%)	14 (66.7%)	0.022
PSL	111 (95.6%)	18 (85.7%)	0.072
Immunomodulator	33 (28.4%)	6 (28.5%)	0.99
Biological	37 (31.9%)	3 (14.2%)	0.102
Admission date:			
CAI on admission	13.0 ± 2.8	13.2 ± 2.8	0.754
Hb on admission (g/dl)	12.2 ± 2.2	11.3 ± 1.4	0.035
Alb on admission (g/dl)	3.4 ± 0.6	3.0 ± 0.5	0.012
CRP on admission (mg/dl)	3.9 ± 5.6	5.0 ± 5.8	0.035
eGFR on admission (ml/min)	89.8 ± 23.5	64.7 ± 14.7	< 0.001
Cr on admission (ml/min)	0.82 ± 0.8	0.8 ± 0.1	0.266
Mayo score on admission	2.8 ± 0.3	2.9 ± 0.2	0.3873
UCEIS score on admission	6.4 ± 1.1	6.7 ± 0.9	0.3704
Time to reach target TAC trough (days)	2.7 ± 1.9	3.2 ± 2.5	0.5879
Length of hospital stay (day)	32.8 ± 12.6	30.7 ± 12.7	0.7335
Concomitant disease:^a^yes	37	56	< 0.0001
Cardiovascular disease (atrial fibrillation/ cardiomyopathy/ hypertension)	4 (0 / 0 / 3)	8 (2 / 0 / 8)	< 0.0001
Gastrointestinal disease (gastric ulcer)	0	1	0.0183
Liver disease (hepatitis B/ hepatitis C, fatty liver/ PSC)	5 (0/1/3/1)	2 (1/0/1/0)	0.26
Pulmonary disease (Asthma,Asthma and COPD Overlap,pneumocystis pneumonia/ interstitial pneumonia)	2 (1 / 0 / 0 / 1 / 0)	4 (1 / 1 / 0 / 0 / 2)	0.0033
Renal disease (Interstitial Nephritis/Renal Transplantation/Diabetic Kidney Disease)	2 (1 / 1 / 0)	1 (0 / 0 / 1)	0.3814
Neurological disease (cerebral infarction/ Parkinson disease/brain artery)	2 (0 / 0 / 2)	3 (1 / 1 / 1)	0.0191
Mental illness (bipolar disorder/ depression/ insomnia)	8 (0/3/5)	8 (1 / 2 / 3)	0.0018
Metabolic disease^a^ (thyroid disease, hyperlipidemia/ hyperuricemia, diabetes)	5 (0/ 5 / 0 / 0)	7 (3/ 2 / 1 / 1)	< 0.0001
Orthopedic disease (osteoporosis/ cervical disc herniation/ spinal canal stenosis)	5 (5 / 0 / 0)	10 (5 / 2 / 3)	0.04
Autoimmune disease (rheumatoid arthritis/ Hashimoto disease/ Takayasu’s disease, systemic lupus erythematosus)	2 (0/ 0 / 1 / 1)	3 (1/ 1 / 1 / 0)	0.0191
Ophthalmologic disease (glaucoma/ cataract)	2 (0 / 2)	5 (2 / 3)	0.0005
Cancer (Uterine cancer Renal cancer Prostate cancer)	0	4 (1 / 1 / 1 / 1)	< 0.0001
CCI	0.42 ± 0.74	1.76 ± 0.94	< 0.0001

Data are the mean ± SD or the number of patients. *CCI* Charlson comorbidity index, *Cr* Creatinine clearance, *CRP* C-reactive protein, *EAI* endoscopic activity index, *eGFR* glomerular filtration rate, *Hb* hemoglobin, *ns* not significant, estimated *UCEIS* Ulcerative Colitis Endoscopic Index of Severity

^a^Some patients had more than one disease

### Remission rate and adverse events

The remission rate was slightly higher in the younger patient group, but the difference was not statistically significant (Table 2). There was also no significant group difference in TAC-induced adverse events (Table 2). All adverse events improved after discontinuation or dose reduction of TAC or intravenous fluid infusion.

**Table 2:** Remission induction rate and side effects

	Younger (< 65 yrs. old) *n* = 116	Elderly (≥65 yrs. old) *n* = 21	*p*-value
Patients achieving remission (%)	93 (80.1%)	14 (66.6%)	0.1862
Total adverse effects of TAC:	40	8	0.7507
*tremor	22	4	0.8426
*Headache	2	2	0.0981
*nausea	1	0	0.5632
*renal impairment	14	4	0.4721
*hyperglycemia	1	0	0.5632

*Some patients had more than one complication

### TAC treatment period and post-treatment renal function

The TAC treatment period was significantly longer in the younger patient group. Before TAC treatment, eGFR was significantly higher in the younger patient group, i.e., renal function was significantly worse in the older patient group.

**Fig. 2:**
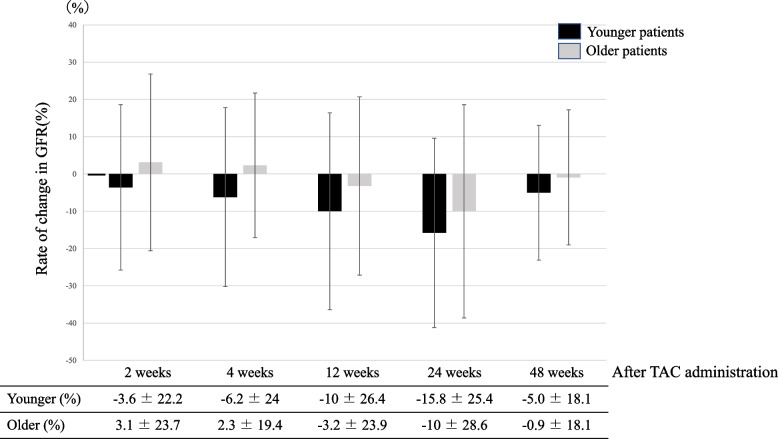
Change in estimated glomerular filtration rate after tacrolimus administration in younger (< 65 years old) and older patients (≥ 65 years old) with moderate to severe ulcerative colitis. The figure shows results at 2, 4, 12, and 24 weeks after initiation of tacrolimus treatment and at 48 weeks after discontinuation of treatment

At weeks 2, 4, 12 and 24 after TAC treatment initiation, eGFR was significantly lower in the older patient group, indicating worse renal function. The same result was found at week 48 after TAC discontinuation. No significant differences between groups were found in the change in eGFR from treatment initiation to week 2, 4, 12, and 24 or to week 48 after TAC discontinuation (Fig. 2, Table 3).

**Table 3:** Duration of tacrolimus administration and renal function

	Younger (< 65 yrs. old) *n* = 110	Elderly (≥65 yrs. old) *n* = 19	*p*-value
TAC administration period (day)	254.4 ± 251.1	156.6 ± 146.6	0.0489
GFR before TAC administration	90.7 ± 22.3	64.7 ± 14.7	< 0.0001
GFR after TAC administration			
After 2 weeks	86.7 ± 28.4	65.5 ± 16.9	0.0002
Percent change in eGFR after 2 weeks of TAC administration	−3.6 ± 22.2	3.1 ± 23.7	0.2107
After 4 weeks	85.4 ± 24.1	65.9 ± 18.6	0.0005
Percent change in eGFR after 4 weeks of TAC administration	−6.2 ± 24	2.3 ± 19.4	0.0971
After 12 weeks	82.9 ± 24.3	61.3 ± 16.4	< 0.0001
Percent change in eGFR after 12 weeks of TAC administration	−10 ± 26.4	−3.2 ± 23.9	0.4459
After 24 weeks	78.2 ± 21.5	59.6 ± 15.8	0.0001
Percent change in eGFR after 24 weeks of TAC administration	−15.8 ± 25.4	−10 ± 28.6	0.1851
eGFR after 48 weeks of discontinuation	83.8 ± 18.5	62.8 ± 12.9	< 0.0001
Percent change after 48 weeks of discontinuation	−5 ± 18.1	−0.9 ± 18.1	0.2752

## Discussion

The study found that before initiation of TAC treatment, Hb, Alb, Cr and eGFR were significantly lower in older than in younger adults with UC and CRP was significantly higher. Older patients were significantly older at disease onset and treatment initiation and had significantly more underlying diseases, but they had less 5-ASA use. There was no difference in disease duration between the groups. The rates of remission and adverse events were similar in younger and older patients. In patients with follow-up data, eGFR was significantly lower at all time points in older adults. However, during the follow-up period, renal function was poor, but there was no difference in the rate of change in the two groups.

### Patient characteristics

Previously, ulcerative colitis was thought to be a disease that affected young people. In recent years, however, the disease has been observed to occur more frequently in the elderly, and its diagnosis and treatment have been the focus of much attention [9].

The percentage of patients allergic to 5-ASA in all age groups increased from 5.3% in 2007-2010 to 9.1% in 2011-2013 and 16.2% in 2014-2016. 5-ASA allergy is increasing [10]. 5-ASA allergy is reported to be associated with a higher operative rate in treatment-responsive patients. Therefore, the presence of 5-ASA allergy is also an important prognostic factor in ulcerative colitis [11].

There are no reports of 5-ASA allergy being more common in the elderly than in younger patients. However, in this study, we found more 5-ASA allergy in the elderly, although we were examining a small number of patients. This is an elderly patient, although the study was conducted in a small number of cases, suggesting that the treatment course may have been poor.

There was a significant difference between the two groups in terms of experience with biologics. Older patients had less experience with biologics than younger patients. In severe cases of ulcerative colitis, younger patients may receive additional medical therapy, such as tacrolimus, if they do not respond well to biologic agents. However, in elderly patients with ulcerative colitis, if the response to prior therapy is inadequate, surgery should be promptly selected for the postoperative course [1]. Therefore, in the present study, we considered that the patients had less experience with biologic agents than younger patients.

In the current study, CAI at admission was not significantly different between the groups, and all patients had moderate or severe UC. Studies have reported finding no difference in symptoms and severity of UC in younger and older patients; however, when older patients have symptoms such as abdominal pain, diarrhea, or melena, UC must be differentiated from infections, drug-induced enteritis, colonic diverticular disease, ischemic enteritis, and colon cancer, which may pose some diagnostic challenges. Health care providers must pay attention to this point when diagnosing UC in older patients [1, 12].

Pre-treatment Hb, Alb, Cr, and eGFR values were lower in the older patient group, which reflects the fact that health generally declines with advancing age. Moreover, relapse and worsening of UC pose a greater risk to the general health of older people than to that of younger people [1].

Pre-treatment CRP was significantly higher in older patients, but no significant difference was observed in the CAI or colonoscopy scores. The clinical characteristics of older adults with UC are reported to be similar to those of young patients [12, 13]. Therefore, because the older patient group had lower Hb levels and higher CRP levels and CAI and colonoscopy scores, the number of patients with severe UC was considered to be higher in the group of older adults. There was no significant difference in the time to reach TAC trough concentration or the duration of hospitalization.

A significant difference was observed in underlying diseases and comorbidities (assessed with the CCI). In particular, none of the younger patients had a history of cancer, but 4 of the older adults did. Aging is a risk factor for cancer [14], so cancer could be expected to be more prevalent in the older patient group. Patients with IBD and a history of cancer are reported to have a two-fold higher risk of developing new or recurrent cancer than those with IBD without a history of cancer, regardless of whether they are taking immunosuppressants [15]. In the natural course of aging, it is well expected that cancer rates will be higher in the older population [16]. The relationship between TAC use and cancer should be considered carefully in older patients. A recent study reported that use of TAC after liver transplantation induced cancer in a dose-dependent manner [17]. However, caution should be exercised when applying that finding directly to patients with UC because the patient backgrounds are different: In that study, many of the patients underwent liver transplantation because of chronic hepatitis, the rate of alcohol and smoking history was higher than in general population, and 10 to 20% of the patients who underwent liver transplantation continued to drink alcohol. Both alcohol and smoking are a risk factor for cancer [18]. It is unlikely that a history of chronic hepatitis, drinking, and smoking are more common in UC patients, but these factors should be evaluated carefully in case of long-term TAC use in older patients.

As seen in the present study, older people tend to have multiple underlying diseases, making treatment more complex than in younger people. Polypharmacy and a higher rate of hospital admissions may result in decreased physical activity in older patients. As physical and cognitive function declines in the elderly, they are more likely to become inactive and depressed. Loss of opportunities to exercise, use their brains to think and communicate can lead to disconnection from society and progress easily to a bedridden state. Therefore, it is important to prevent physical inactivity in the elderly [19].

A higher CCI is considered to be related to short-term mortality risk: When CCI was compared between younger and older people, patients with a higher CCI were reported to have a higher incidence of drug-induced adverse events [8]. However, although the present study found a significant difference in CCI between the younger and older patient groups, adverse events were not significantly different (see below).

### Remission rate and adverse events associated with TAC

The remission rate with TAC was 80.1% in the younger patient group and 66.6% in the older patient group, but the difference was not statistically significant. A Cochrane review found a remission rate with TAC of about 62%, which was lower than that in both age groups in the present study [20]. In general, the remission rate with biologics is also reported to be lower in older adults [21]. The TAC was not significantly different, but it was suggested that the effect may be less effective in the elderly group. However, in this study, the remission rate was better in the elderly group than previously reported. Therefore, we believe that TAC is an effective treatment for the elderly as well.

Adverse events related to TAC were not significantly different between the groups. These adverse events were already reported elsewhere [22]. All adverse events improved after discontinuation or dose reduction of TAC or intravenous fluid infusion.

### TAC treatment period and post-treatment renal function

The TAC treatment period was significantly different between the groups. The Japanese Clinical Guideline recommends a TAC treatment period of about 90 days, whereas the European Crohn’s and Colitis Organisation guideline recommends 180 days [3, 23]. At our hospital, TAC is used until remission or mucosal healing because the relapse rate is lower after mucosal healing [24]. Therefore, the duration of TAC treatment is generally longer at our hospital than is recommended in treatment guidelines. There have been reports from Japan of increased efficacy with tacrolimus use for longer than 90 days [25].

The duration of TAC treatment was significantly shorter in the older patients, which is hypothesized to be due to the higher risk of adverse events. For example, patients with an eGFR lower than 30% are reported to be more likely to develop chronic nephropathy. Accordingly, renal function tests need to be conducted in a timely manner [26]. With regard to renal function before and after TAC treatment, the older patient group had a significantly lower eGFR at all time points. Aging is a risk factor for deterioration of renal function [27]. However, we found no difference in the change in eGFR between the groups. In fact, eGFR declined less in the older patients up to 4 weeks after TAC treatment initiation. Patients are likely to be hospitalized when TAC treatment is initiated, and during hospitalization, older patients in particular tend to receive fluid infusions to support kidney function, which may explain why renal function was preserved in the older patients. The underlying mechanism of TAC-induced renal impairment is contraction of blood vessels, which results in decreased renal blood flow via renal arterioles [28]. Fluid infusion increases renal blood flow volume and protects the kidneys. Especially in older patients in whom cardiovascular stress is not an issue, fluid infusion is recommended during treatment with TAC.

Our results indicate that TAC is a treatment option for older patients with moderate to severe UC. However, in addition to renal function, attention must be paid to the aforementioned cancer risk and to infection related to reduced immunity because aging is a risk factor also for infections [29]. During treatment with TAC, older adults must be carefully monitored for signs of infection, including physical findings, such as fever and tachycardia, results of blood tests, and diagnostic imaging. If these parameters are carefully followed, TAC is an effective drug and is an important treatment option for moderate and severe UC in older patients.

The limitations of this study are that it was retrospective and performed at a single study site. Consequently, bias can be expected. A prospective, multi-center study should be performed in a larger number of patients.

## Conclusions

TAC can be used safely in elderly patients with moderate to severe UC with careful monitoring.

## Data Availability

Data used/ analyzed during the current study are available from the Corresponding Author on reasonable request.

## Abbreviations

*5-ASA*: 5 acetylsalicylic acid
*Alb*: albumin
*AZA*: Azathioprin
*CAI*: Clinical active index
*CCI*: Charlson Comorbidity Index
*COPD*: chronic obstructive pulmonary disease
*Cr*: Creatinine clearance
*CRP*: C-reactive protein
*EAI*: endoscopic activity index
*eGFR*: estimated glomerular filtration rate
*Hb*: hemoglobin
*TAC*: Tacrolimus
*PSL*: Prednisolone
*UC*: Ulcerative colitis
*UCEIS*: ulcerative colitis endoscopic index of severity
